# Supplementary material for: Mutations in PCYT2 disrupt etherlipid biosynthesis and cause a complex hereditary spastic paraplegia
Source: Brain. 2019 Oct 22;142(11):3382–97. doi: 10.1093/brain/awz291 (PMC6821184; doi:10.1093/brain/awz291)
Supplement: awz291_Supplementary_Data [file awz291_supplementary_data.zip › awz291-Suppl_data/Supplementary_Data4.zip!/Supplementary information 3 - fibroblast lipidomics.zip!/Fibroblast lipidomics/Multiple comparison/PCA.pdf]

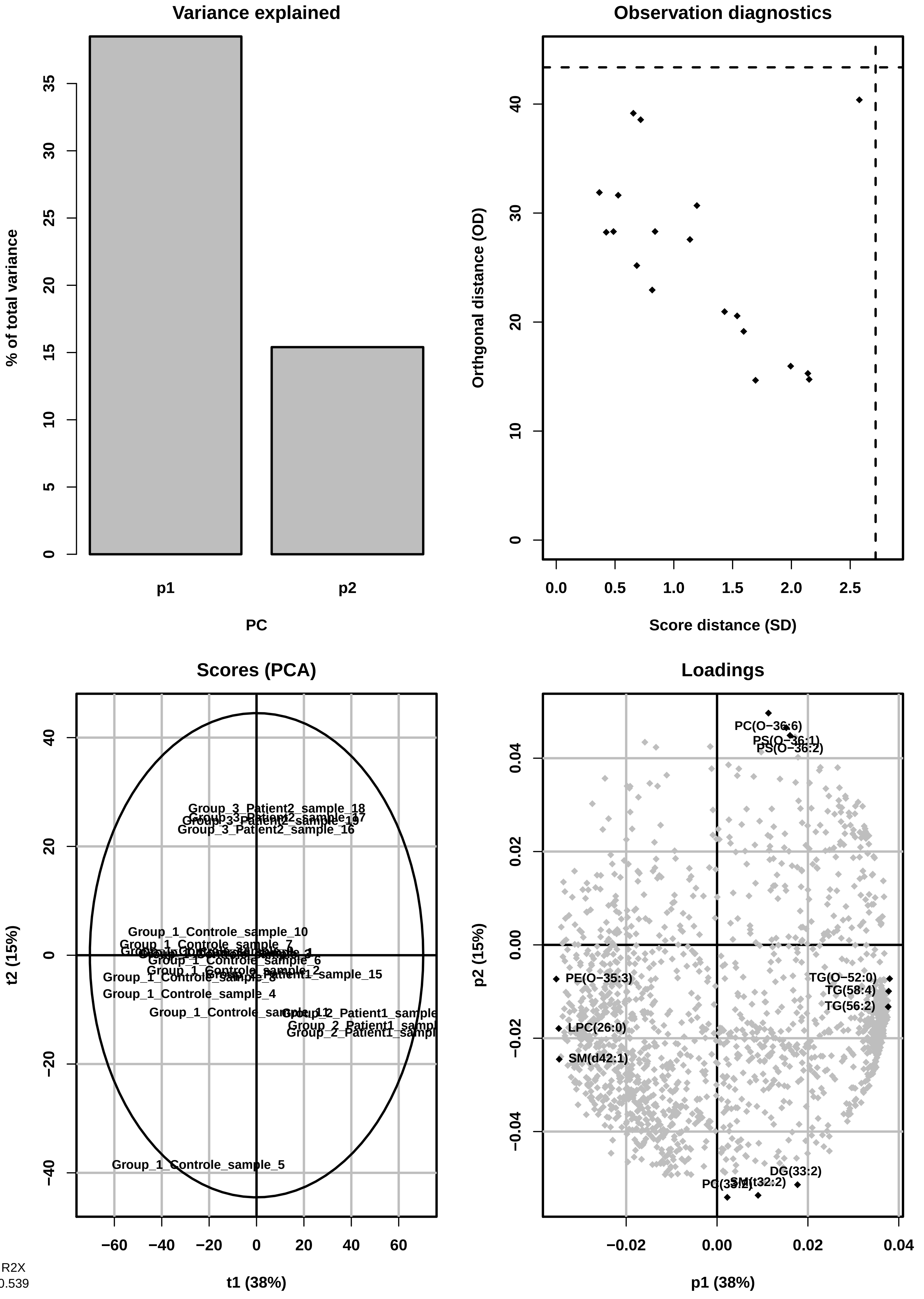

Variance explained
% of total variance
0
5
10
15
20
25
30
35
p1
p2
PC
Observation diagnostics
Orthgonal distance (OD)
0
10
20
30
40
0.0
0.5
1.0
1.5
2.0
2.5
Score distance (SD)
Scores (PCA)
t2 (15%)
40
20
0
−20
−40
−60
−40
−20
0
20
40
60
t1 (38%)
Group_3_Patient2_sample_18
Group_3_Patient2_sample_16
Group_1_Controle_sample_10
Group_1_Controle_sample_7
Group_1_Controle_sample_6
Group_1_Controle_sample_2
Group_1_Controle_sample_8
Group_1_Controle_sample_4
Group_1_Controle_sample_5
Loadings
p2 (15%)
0.04
0.02
0.00
−0.02
−0.04
−0.02
0.00
0.02
0.04
p1 (38%)
PC(O−36:6)
PS(O−36:1)
PS(O−36:2)
PE(O−35:3)
LPC(26:0)
SM(d42:1)
TG(O−52:0)
TG(58:4)
TG(56:2)
DG(33:2)
R2X
0.539
